# Supplementary material for: Plasma taurine level is linked to symptom burden and clinical outcomes in post-COVID condition
Source: PLoS One. 2024 Jun 5;19(6):e0304522. doi: 10.1371/journal.pone.0304522 (PMC11152273; doi:10.1371/journal.pone.0304522)
Supplement: Attachment — Submitted filename: Response to the Editor and Reviewers.docx [file pone.0304522.s003.docx]

**Response to the editor:**

**1. Please ensure that your manuscript meets PLOS ONE's style requirements, including those for file naming. The PLOS ONE style templates can be found at** 
[**https://journals.plos.org/plosone/s/file?id=wjVg/PLOSOne_formatting_sample_main_body.pdf**](https://journals.plos.org/plosone/s/file?id=wjVg/PLOSOne_formatting_sample_main_body.pdf) **and** [**https://journals.plos.org/plosone/s/file?id=ba62/PLOSOne_formatting_sample_title_authors_affiliations.pdf**](https://journals.plos.org/plosone/s/file?id=ba62/PLOSOne_formatting_sample_title_authors_affiliations.pdf)**.**

**Response:** We would like to thank the editor for their effort towards our manuscript. We made sure that our manuscript meets the style requirements found in the links.

**Please state what role the funders took in the study.  If the funders had no role, please state: "The funders had no role in study design, data collection and analysis, decision to publish, or preparation of the manuscript." If this statement is not correct you must amend it as needed. 
Please include this amended Role of Funder statement in your cover letter; we will change the online submission form on your behalf.**

**3. Thank you for stating the following in the Acknowledgments Section of your manuscript: 
"This work is supported by grants from the Canadian Institutes of Health Research (grant no. PJT-451105), the Northern Alberta Clinical Trials and Research Centre (grant no. RES50821), and the long COVID web (grant no. RES0065210) at the University of Alberta."**

**We note that you have provided funding information that is not currently declared in your Funding Statement. However, funding information should not appear in the Acknowledgments section or other areas of your manuscript. We will only publish funding information present in the Funding Statement section of the online submission form. 
Please remove any funding-related text from the manuscript and let us know how you would like to update your Funding Statement. Currently, your Funding Statement reads as follows: 
"For this study, GYO is supported by grants from the Canadian Institutes of Health Research (grant no. PJT-451105), the Northern Alberta Clinical Trials and Research Centre (grant no. RES50821), and the long COVID web (grant no. RES0065210) at the University of Alberta."**

**Please include your amended statements within your cover letter; we will change the online submission form on your behalf.**

**Response:** We thank the editor for this comment. We removed the funding-related text from the acknowledgement section. This section reads as follows now: “We would like to thank the patients, their families, and the dedicated clinical staff and frontline workers at the University of Alberta's COVID-19 units, whose collaboration made this work possible. Special appreciation goes to Dr. Bruce Ritchie and the Canadian Biosample Repository team for their noteworthy efforts in contributing to the CoCollab COVID-19 Study. The metabolomics assays were carried out by The Metabolomics Innovation Center in Edmonton, AB, Canada.”

Please update our funding statement section to this text:

For this study, GYO is supported by grants from the Canadian Institutes of Health Research (grant no. PJT-451105), the Northern Alberta Clinical Trials and Research Centre (grant no. RES50821), and the Long COVID web (grant no. RES0065210) at the University of Alberta.

**4. Please include captions for your Supporting Information files at the end of your manuscript, and update any in-text citations to match accordingly. Please see our Supporting Information guidelines for more information:** [**http://journals.plos.org/plosone/s/supporting-information**](http://journals.plos.org/plosone/s/supporting-information)**.**

**Response:** We thank the editor for this comment. We have added the caption for supporting information file at the end of our manuscript.

**Changes made:**

Supporting information

S1 File. Supporting data values of all plots.

**5. Please review your reference list to ensure that it is complete and correct. If you have cited papers that have been retracted, please include the rationale for doing so in the manuscript text, or remove these references and replace them with relevant current references. Any changes to the reference list should be mentioned in the rebuttal letter that accompanies your revised manuscript. If you need to cite a retracted article, indicate the article’s retracted status in the References list and also include a citation and full reference for the retraction notice.**

**Response:** We ensured that all references used in this manuscript are complete and correct. Vancouver style used for this section as requested in the author’s guideline. We also made sure that there is no retracted article cited.

**While revising your submission, please upload your figure files to the Preflight Analysis and Conversion Engine (PACE) digital diagnostic tool,** [**https://pacev2.apexcovantage.com/**](https://pacev2.apexcovantage.com/)**. PACE helps ensure that figures meet PLOS requirements. To use PACE, you must first register as a user. Registration is free. Then, login and navigate to the UPLOAD tab, where you will find detailed instructions on how to use the tool. If you encounter any issues or have any questions when using PACE, please email PLOS at** [**figures@plos.org**](mailto:figures@plos.org)**. Please note that Supporting Information files do not need this step.**

**Response:** We have checked all the figures by Preflight Analysis and Conversion Engine (PACE) to ensure they all meet the PLOS requirements.

**Response to reviewers:**

**Reviewer #1: Authors should provide data which can support the taurine administration and its benefits like reduced depressive behavior, improved memory, and mitigation of age-related issues.**

**Response:** We would like to thank the reviewer for their time and effort towards our manuscript. As we have elaborated in the discussion section, taurine administration enhances various biological processes, resulting in beneficial neurological functions, such as reduced depression, improved memory, and alleviation of age-related issues. We have now cited several references that show taurine supplementation could enhance the cognitive function in different disorders. This is consistent with our findings where plasma taurine was positively correlated with different biomarkers involved in those biological processes and negatively associated with neurological symptoms in patients with post-COVID condition. Please note that in this study, we just examined the plasma taurine levels in patients with PCC during the acute and convalescence phase, and we did not supplement the patients with taurine. At the end of the discussion, we also mentioned that a large clinical trial is required to test these beneficial functions of taurine in PCC patients directly.

**Changes made:**

**Page 16, line 323 (Discussion): “**Similar beneficial effects have also been observed in clinical studies, where taurine supplementation significantly improved the cognitive function of patients with dementia, first-episode psychotic disorder, and Mitochondrial encephalomyopathy with lactic acidosis and stroke-like episodes (MELAS).(23-25) Consistently, our study showed higher plasma taurine level was correlated with various biomarkers reflecting improved physiological states, along with negative associations with different self-reported PCC symptoms, such as weakness, cognitive impairment, and mood disturbance.”

**The authors are expected to study the correlation between the plasma taurine levels and therapeutic activities, including antioxidant, anti-aging, antiepileptic, cytoprotective, and cardioprotective effects in patients having diseases like diabetes, obesity. It will provide a scope for researches in this domain in future.**

**Response:** We thank the reviewer for this comment. We agree that taurine can have more potent beneficial effects on PCC patients who are comorbid with other diseases, such as diabetes, obesity, or hypertension. To evaluate this, we assessed plasma taurine levels in patients with other comorbidities during the convalescence phase of COVID-19 disease. Out of all the comorbidities reported in Table 2, we could find that plasma taurine levels were reduced in PCC patients with diabetes compared to PCC patients without diabetes. We also demonstrated that taurine significantly correlates with other molecules with antioxidation, anti-inflammation, and cryoprotective properties in PCC patients with diabetes. These findings are now presented in the result and discussion sections.

**Changes made:**

**Page 14, line 283 (Results, section 6): “Plasma taurine levels in patients with diabetes**

Plasma taurine levels were examined in PCC patients with diabetes during the convalescence phase. Notably, PCC patients with diabetes exhibited reduced plasma taurine concentrations compared to PCC patients without diabetes (p<0.05) (Fig 6A). Further exploration of plasma samples from PCC patients with diabetes revealed associations between taurine and several key biomarkers involved in different biological functions. Plasma taurine levels were positively correlated with anti-inflammatory cytokines, namely interleukin-4 (R=0.51, p<0.001) and interleukin-13 (R=0.4, p=0.0046) (Fig 6B-C). Similarly, plasma taurine levels positively correlated with antithrombin-3 (R=0.38, p=0.0064) and peroxiredoxin-1 (R=0.62, p<0.001), which are involved in the inhibition of coagulation and oxidative stress, respectively (Fig 6D-E). Moreover, plasma taurine levels showed a positive correlation with apolipoprotein A1 (APO A-I) (R=0.29, p=0.043) in PCC patients with diabetes, which illustrates a potential role for taurine in lipid metabolism and cholesterol balance (Fig 6F). Moreover, we found that plasma taurine levels were positively associated with creatine (R=0.32, p=0.026), a source of energy in the body (Fig 6G). Lastly, taurine levels were negatively correlated with quinolinic acid (R=-0.3, p=0.038), a neurotoxic breakdown metabolite of tryptophan (Fig 6H).

**Fig 6. Plasma taurine alteration and correlations with biomarkers in PCC patients with diabetes.**

(A) Box and whisker plot illustrating the level of taurine in PCC patients with and without diabetes during the convalescence phase compared with healthy controls. Mann-Whitney U test with Benjamini-Hochberg correction was performed for pairwise comparison. (B-H) Scatter plots showing the correlations between plasma taurine levels with IL-4 (B), IL-13 (C), antithrombin III (D), peroxiredoxin I (E), APOA-I (F), creatine (G), and quinolinic acid (H) in PCC patients with diabetes. IL-4, Interleukin-4; IL-13, Interleukin-13; APOA-I, apolipoprotein A1. *p<0.05, **p<0.01, ***p<0.001, ****p<0.0001.”

**Page 16, line 332 (Discussion): “**Many clinical trials have demonstrated the beneficial effects of taurine supplementation for patients with diabetes by improving glycemic indices and lipid profile. (29-33) Our study illustrated that PCC patients with comorbid diabetes had lower taurine levels compared to PCC patients without diabetes. However, taurine levels in these patients were positively correlated with molecules with anti-oxidation, anti-inflammatory, and cytoprotective properties. As such, taurine supplementation may be an effective adjuvant therapy for PCC patients with concurrent diabetes.”

**Authors can contribute some substantial findings to support the taurine supplementation to reduce the total cholesterol and triglycerides in patients with obesity.**

**Response:** As discussed in the previous comment, we cannot directly show that taurine supplementation will reduce total cholesterol and triglycerides in patients with obesity since we did not supplement our patients with taurine. We were able to examine the relationship between changes in cholesterol and triglyceride levels with taurine during follow-up. However, as evident by the figures below, we did not observe a significant difference in plasma taurine levels with and without obesity, which can result from the limited study cohort size. This point was explained in the discussion section to highlight the beneficial effects of taurine in other diseases through various biological functions, which can also help patients with post-COVID condition.

**Figure 1. Plasma Taurine levels in PCC patients during convalescence.**

**(A). Plasma taurine levels in healthy controls, PCC patients with obesity (BMI>30), and PCC patients without obesity (BMI<30).**

**(B). Plasma taurine levels in healthy controls, diabetic obese PCC patients, and PCC patients without obesity and diabetes.**

**Reviewer #2:**

**This study aimed to investigate the role of taurine and possible recommendations of taurine supplementation for post-COVID conditions. Although the manuscript is well written, the results sections were quite confusing for readers. For example, the authors explained the data with repetition (“Fig 1. Taurine in acute and convalescence phase of SARS-CoV-2 infection.” Fig 2. Taurine correlations with biomarkers of inflammation and gut dysbiosis). This must be rewritten.**

**Response:** We thank the reviewer for their valuable input and comments. In the revised manuscript, we have now separated these two figures into two different sections in the result to offer more clarity for readers. We also rewrote some sentences in this part of the manuscript so that it will read better.

**Changes made:**

**Page 10, line 184 (Results, section 2):** “**Temporal changes in taurine levels following SARS-CoV-2 infection**

Metabolomic analysis of plasma samples in the convalescence phase identified 50 differentially expressed metabolites compared to age and sex-matched healthy controls, with the amino acid taurine as one of the top upregulated metabolites (fold-change=5.7, p<0.0001) (Fig 1B). Additionally, plasma taurine levels were significantly elevated in acute infection compared to healthy controls (p=1.1e-15) and were further elevated in the convalescence phase compared to the acute phase (p=1.8e-11) (Fig 1C). Taurine could protect against PCC through inhibition of various pathophysiological processes and stimulation of beneficial functions (Fig 1D). Therefore, we hypothesize that failure to upregulate the taurine pathway may predispose individuals to having adverse outcomes and greater symptom burden during convalescence from SARS-CoV-2 infection.”

**Page 11, line 202 (Results, section 3):** “**Correlation between taurine levels with inflammatory and microbiome biomarkers in post-COVID condition**

We examined the associations between plasma taurine levels and biomarkers of pathophysiological processes identified in PCC during the convalescence phase. We found that plasma levels of taurine negatively correlated with markers of inflammation, such as C-reactive protein (R=-0.22, p=0.019), interleukin-10 (R=-0.23, p=0.014), and interleukin-6 (R=-0.28, p=0.002) (Fig 2A-C). In contrast, there was a positive correlation between taurine levels with tryptophan (R=0.29, p=0.001) and serotonin (R=0.4, p=8.8e-6), while demonstrating a strong negative correlation with quinolinic acid (r=-0.4, p<0.001), a byproduct of tryptophan metabolism with neurotoxic effects (Fig 2D-F). Lastly, taurine was negatively correlated with biomarkers related to gut dysbiosis, such as trimethylamine N-oxide (TMAO) (R=-0.27, p=0.003), phenylacetic acid (R=-0.22, p=0.016), and lipopolysaccharide binding protein (R=-0.27, p=0.0031), suggesting a possible role of taurine in modulating microbial ecology in PCC (Fig 2G-I).”

**Correlation between taurine levels with inflammatory and microbiome biomarkers in post-COVID condition**

We examined the associations between plasma taurine levels and biomarkers of pathophysiological processes identified in PCC during the convalescence phase. We found that plasma levels of taurine negatively correlated with markers of inflammation, such as C-reactive protein (R=-0.22, p=0.019), interleukin-10 (R=-0.23, p=0.014), and interleukin-6 (R=-0.28, p=0.002) (Fig 2A-C). In contrast, there was a positive correlation between taurine levels with tryptophan (R=0.29, p=0.001) and serotonin (R=0.4, p=8.8e-6), while demonstrating a strong negative correlation with quinolinic acid (r=-0.4, p<0.001), a byproduct of tryptophan metabolism with neurotoxic effects (Fig 2D-F). Lastly, taurine was negatively correlated with biomarkers related to gut dysbiosis, such as trimethylamine N-oxide (TMAO) (R=-0.27, p=0.003), phenylacetic acid (R=-0.22, p=0.016), and lipopolysaccharide binding protein (R=-0.27, p=0.0031), suggesting a possible role of taurine in modulating microbial ecology in PCC (Fig 2G-I).

**Page 10, line 190 (Results, section 2):** “~~Taurine can protect PCC patients from various pathophysiological events driving it (Fig 1D), and therefore, variation in the trajectory of plasma taurine levels can provide important insight into the PCC.~~ Taurine could protect against PCC through inhibition of various pathophysiological processes and stimulation of beneficial functions (Fig 1D). Therefore, we hypothesize that failure to upregulate the taurine pathway may predispose individuals to having adverse outcomes and greater symptom burden during convalescence from SARS-CoV-2 infection.”

**Page 11, line 208 (Results, section 3):** “~~In contrast, there was a positive correlation between taurine levels and biomarkers of tryptophan regulation, including tryptophan itself (R=0.29, p=0.001) (Fig 2D), and serotonin (R=0.4, p=8.8e-6) (Fig 2E).~~ In contrast, there was a positive correlation between taurine levels with tryptophan (R=0.29, p=0.001) and serotonin (R=0.4, p=8.8e-6), while demonstrating a strong negative correlation with quinolinic acid (r=-0.4, p<0.001), a byproduct of tryptophan metabolism with neurotoxic effects (Fig 2D-F).

**The authors explained about gut dysbiosis. However, it needs to be explained with the taurine supplementation with gut dysbiosis markers in the PCC.**

**Response:** We appreciate the reviewer for mentioning this point. Many studies show that taurine can alleviate gut dysbiosis and its consequences, including gut permeability, by enhancing gut composition and tight junction proteins, as cited in the discussion section. Taurine supplementation efficacy has yet to be tested in the PCC. However, it has been assessed for other diseases, so we proposed a large-scale clinical trial that would enable researchers to examine taurine's beneficial effects on PCC patients thoroughly. This is consistent with our findings where plasma taurine was negatively correlated with gut-derived metabolites, such as Trimethylamine N-oxide (TMAO), quinolinic acid, and phenylacetic acid (**Fig 2F-H**). These plots are now added to figure 2 to highlight this potential of taurine to alleviate the consequences of gut dysbiosis.

**Changes made:**

**Page 11, line 208 (Results, section 3):** “In contrast, there was a positive correlation between taurine levels with tryptophan (R=0.29, p=0.001) and serotonin (R=0.4, p=8.8e-6), while demonstrating a strong negative correlation with quinolinic acid (r=-0.4, p<0.001), a byproduct of tryptophan metabolism with neurotoxic effects (Fig 2D-F). Lastly, taurine was negatively correlated with biomarkers related to gut dysbiosis, such as trimethylamine N-oxide (TMAO) (R=-0.27, p=0.003), phenylacetic acid (R=-0.22, p=0.016), and lipopolysaccharide binding protein (R=-0.27, p=0.0031), suggesting a possible role of taurine in modulating microbial ecology in PCC (Fig 2G-I).”

**Page 17, line 345 (Discussion):** “Gut dysbiosis has been identified as another pathophysiological mechanism in the PCC. (2, 35) Emerging evidence found alterations in gut microbiome of patients with PCC, resulting in the accumulation of gut-derived metabolites in the systemic circulation and resultant clinical manifestation of gastrointestinal and neurological symptoms. (1, 36) Taurine alleviates these conditions through different mechanisms, including regulation of gut microbiota composition and enhancing tight junction proteins, thereby reducing gut permeability. (37-40) In accordance, we found that plasma taurine levels were negatively correlated with gut-derived metabolites, such as TMAO, quinolinic acid, and phenylacetic acid in PCC patients during convalescence.”

**In addition, authors explaining about the role of taurine and tryptophan could strengthen these results.**

**Response:** We thank the reviewer for this comment. We agree that highlighting the link between taurine and tryptophan will strengthen our manuscript since recent studies have found that tryptophan absorption is dysregulated in PCC patients, thus reducing serotonin levels. We have now added to the discussion of this finding and showed that plasma taurine positively correlates with tryptophan and serotonin in our cohort during the convalescence period.

**Changes made:**

**Page 17, line 353 (Discussion):** “Another pathophysiological link between PCC and gut function is through tryptophan dysregulation. PCC is associated with decreased intestinal absorption of serotonin precursor, tryptophan, which impacts downstream serotonin metabolism and availability, possibly explaining certain neurocognitive symptoms in PCC patients. (41) Interestingly, taurine has a positive effect on serotonin levels, which has been demonstrated in both in vitro and in vivo studies. (42, 43) Consistent with these findings, plasma taurine levels in PCC patients were positively correlated with serotonin concentration and its precursor tryptophan during convalescence.”
